# 2287. Goal! Goal! Goal! Detection of SARS-CoV-2 Variants in Travelers During the FIFA World Cup, Qatar 2022 — CDC Traveler-Based Genomic Surveillance Program, November 2022–January 2023

**DOI:** 10.1093/ofid/ofad500.1909

**Published:** 2023-11-27

**Authors:** Katrina M Byrd, Stephen Bart, Teresa C Smith, Samantha M Loh, Sarah Anne J Guagliardo, Benjamin Rome, Thomas Aichele, Ezra T Ernst, Robert C Morfino, Martin S Cetron, Cindy R Friedman, Allison T Walker

**Affiliations:** Centers for Disease Control and Prevention, Atlanta, Georgia; Centers for Disease Control and Prevention, Atlanta, Georgia; CDC, Atlanta, Georgia; Centers for Disease Control and Prevention, Atlanta, Georgia; Centers for Disease Control and Prevention, Atlanta, Georgia; Ginkgo Bioworks, Inc, Boston, Massachusetts; Ginkgo Bioworks, Inc, Boston, Massachusetts; XpresCheck, Moorestown, New Jersey; Ginkgo Bioworks Inc, Winthrop, Massachusetts; Centers for Disease Control and Prevention, Atlanta, Georgia; CDC, Atlanta, Georgia; Centers for Disease Control and Prevention, Atlanta, Georgia

## Abstract

**Background:**

Over 1 million people traveled to Qatar and surrounding countries during the World Cup 2022 soccer tournament, a mass gathering that could enhance transmission of SARS-CoV-2 variants. Minimal information about circulating variants in World Health Organization (WHO) Eastern-Mediterranean region (EMRO) which includes Qatar, United Arab Emirates, and Saudi Arabia were available from global data bases (GISAID). The Traveler-based Genomic Surveillance (TGS) program samples arriving international travelers to detect new variants and fill surveillance gaps. Monitoring of flights from EMRO was increased during the tournament.

**Methods:**

Travelers at six US airports volunteered to provide nasal swabs that were pooled by flight origin and sent to laboratories for SARS-CoV-2 reverse transcription polymerase chain reaction. Positives underwent whole genome sequencing. Pool positivity and variant frequencies were compared for travelers from EMRO vs. other regions. Chi-square tests were used to determine statistical significance.

**Results:**

During November 20, 2022–January 2, 2023, 2050 sample pools from 16,595 travelers were collected. Of these, 100 pools (703 travelers) were from EMRO and 1950 pools (15,892 travelers) were from other regions. Pool positivity from EMRO was 28% (28/100) vs. 25% (489/1950) in other regions (p=0.51). Regionally detected variant proportions were: EMRO (n=25) XBB (40%), BQ.1 (20%), BQ.1.1 (20%); Europe (n=166) BQ.1.1 (48%), BQ.1 (20%), XBB (9%); Western Pacific (n=51) BA.5 (24%), BN.1 (22%), XBB (18%); South-East Asia (n=21) BQ.1.1 (33%), XBB (29%), BA.2 (10%), BA.5 (10%), XBB.1.5 (10%); and Africa (n=15) BQ.1.1 (53%), BA.5 (27%), XBB (20%). The proportion of XBB in EMRO was significantly higher than Europe and Western Pacific regions (p< 0.05).

**Conclusion:**

During World Cup 2022, SARS-CoV-2 positivity was similar for travelers from EMRO compared to all other regions. Variant proportions differed among regions, with EMRO having the highest proportion of XBB, a recombinant lineage associated with higher transmissibility. TGS fills gaps in SARS-CoV-2 surveillance and can be surged during mass gathering events.

**Disclosures:**

**Benjamin Rome, MBA**, Ginkgo Bioworks, Inc.: Employee|Ginkgo Bioworks, Inc.: Stocks/Bonds **Robert C. Morfino, MBA**, Ginkgo Bioworks Inc.: I am an employee|Ginkgo Bioworks Inc.: Stocks/Bonds

